# Pharmacy students' perceived willingness and ability to negotiate for paid co-operative education positions

**DOI:** 10.1016/j.rcsop.2021.100026

**Published:** 2021-05-15

**Authors:** Brenda Y. Oh, Richard Violette, Kelly A. Grindrod, Nancy M. Waite, Sherilyn K.D. Houle

**Affiliations:** a School of Pharmacy, University of Waterloo, 200 University Avenue West, Waterloo, Ontario N2L 3G1, Canada; b School of Human Services and Social Work, Griffith University, 170 Kessels Road, Nathan, QLD 4111, Australia

**Keywords:** Pharmacy students, Gender, Negotiation, Salaries, Employment

## Abstract

**Background:**

Evidence of a gender wage gap has been identified across many professions, with some evidence in pharmacy. Negotiation is one potential strategy to address this gap and it is underutilized, especially among women. No studies to date have examined pharmacy student perceptions of negotiation when applying for co-operative education positions – a potential sign of willingness to negotiate for entry-level positions upon graduation.

**Objectives:**

To examine pharmacy students' comfort with and ability to engage employers in negotiation over wage and other work-related considerations for mandatory and paid co-operative education work terms at the University of Waterloo (Waterloo, Ontario, Canada).

**Methods:**

Two focus groups, one for female and one for male students, were performed with students who had completed at least one co-operative education placement. Focus groups aimed to elucidate students perceived ability to negotiate with potential employers, to identify strategies that educators can employ to better support students through the hiring and negotiation process, and to elicit student perceptions on the gender wage gap in pharmacy. Focus groups were audio recorded and transcribed verbatim, and data were coded inductively by two independent reviewers, employing thematic analysis.

**Results:**

Three major and two minor themes were identified: Preservation of the relationship; Power differential and perceived ability to negotiate; Institutional support and training; Negative experience with negotiation and wage gaps; and the presence of a gendered approach to negotiation. Pharmacy students rarely engage in negotiation during co-operative hiring processes at the University of Waterloo, with some female students expressing hesitation to negotiate due to concerns about being perceived as “bossy”. Students of both genders felt poorly equipped to engage in negotiation with a potential employer, and lacked confidence in initiating such a conversation.

**Conclusions:**

Students identified a number of factors which influence their ability and desire to negotiate wages during co-op placement, including the requirements and logistics of placements, the perceived power imbalance between students and potential employers, and a concern that any wage negotiation may overshadow the value placed by students on the opportunities to provide patient care. Educators can play a role in equipping students, especially female students, with tools to enter into conversations on wages with potential employers.

## 1 Introduction

Gender inequity remains a pervasive and persistent issue affecting women around the world. The World Economic Forum's 2018 Global Gender Gap Report states that, on average, women face a 32% disparity in measures of economic participation, educational attainment, health and survival, and political empowerment.^1^ In particular, the greatest disparities exist in the domains of political empowerment and economic participation and opportunity – the latter of which includes wages and career advancement.^1^

Evidence of a gender wage gap has been reported in a number of health professions, including medicine,^2^, ^3^, ^4^ nursing^5^, ^6^, ^7^ and optometry.^8^ However, there has been limited large-scale exploration of a gender wage gap in pharmacy. A survey of over 1400 pharmacists in South Florida identified the presence of gender bias after controlling for a number of factors. This survey found that men earned significantly more, with a mean annual salary of $102,550 versus $95,054 for women.^9^ Commonly cited factors that potentially contribute to gender wage inequity such as hours worked per week, level of education, and having children did not appear to disadvantage women in this study. Hours worked per week were similar (average 39.6 h for men vs. 38.5 for women), women were almost twice more likely to have a PharmD degree than men, and the average number of children in the household was 1.7 among men and 1.2 among women (although the study did not assess the amount of time spent in a childcare capacity). In this study, men had more years of experience at 27.8 years vs. 14.7 years for women, which may have some impact on wages realized; however, the authors concluded that systemic bias could not be ruled out.

A number of strategies have been proposed to identify and address gender-based wage gaps, such as salary transparency, and regular auditing, reporting, and correcting of wage differences through a like-for-like pay model.^10^ However, such approaches are largely reactive in nature and seek to correct inequities already in place. Equipping women with skills in negotiation may be seen as a more proactive strategy. Indeed, Kolb proposes that “women can take actions to remedy these situations, and one of them is to negotiate more proactively and effectively for wages and opportunity”.^11^ However, research has found that women may internalize gender stereotypes when approaching negotiation, particularly stereotypes related to women being less self-interested and assertive than men, and may lower their aspirations out of a fear of backlash as a result.^12^^,^^13^ Even small differences in negotiation outcomes can become amplified over time, as future negotiation and annual wage increases, for example, will depend on the degree of success during previous negotiations. Gender-based wage differentials may begin early in one's career, as evidenced by research identifying a wage gap exceeding USD $16,000 per year among new male versus female medical graduates.^2^ Thus, equipping trainees, particularly women, to negotiate may help to ensure that all new practitioners are able to earn adequate and equitable wages.

Currently, only two pharmacy programs in North America offer co-operative (co-op) education as a form of work-integrated learning – one of which is the University of Waterloo in Waterloo, Ontario, Canada.^14^ In the co-op model at Waterloo, students apply and interview for, and are hired into, paying jobs for their mandatory experiential education during their second and third years. After interviewing, both students and employers rank their preferred position and candidate, respectively, and are matched using a computer algorithm. As students are paid employees for these experiential education experiences, this model provides the opportunity to study how these students approach the hiring process very early in their careers, and identify strategies educators can consider to best prepare them to enter the workforce as pharmacists. The objective of this study is therefore to examine students' readiness and willingness to negotiate for co-op positions, and to examine student perceptions of the existence of a gender wage gap in the pharmacy profession.

## 2 Methods

### 2.1 Study design

A qualitative study consisting of two focus groups was conducted. Reporting of the study design and findings is in accordance with the consolidated criteria for reporting qualitative research (COREQ).^15^

### 2.2 Participants and recruitment

Pharmacy students at the University of Waterloo School of Pharmacy who had completed at least one co-operative learning placement at the time of the study were invited to participate in focus groups via a post to their cohort's respective Facebook page, with each cohort comprising approximately 120 students, and 3 cohorts being eligible to participate. Convenience sampling was utilized to recruit eight male and eight female students for participation. One male student was unable to participate due to scheduling miscommunication. Students were remunerated $35 CAD for their time participating in the focus group.

### 2.3 Preparation for the focus groups

A semi-structured interview guide featuring open-ended questions was developed collaboratively by two authors (B.Y.O. and R.V.). The key aims were to elucidate the student's perceived ability to negotiate with potential employers, and to identify strategies that educators can employ to better support students through the hiring and negotiation process. A secondary aim was to elicit student perceptions on the gender wage gap in pharmacy. The remaining authors reviewed the guide prior to the first focus group. As the focus groups were performed on the same day, modifications of the guide between groups was not feasible; however, differing prompts were employed depending on responses obtained. The interview guide (provided in Appendix 1) was not pilot tested prior to the first focus group.

### 2.4 Data collection

Data collection took place in January 2019. Prior to the focus group, all participants completed an anonymous demographic survey to collect information related to pharmacy class cohort, age, and wages earned at each of their co-op placements, and were informed of the research question and the objectives of the research, and that it was part of an Independent Studies research elective course project by a fourth-year student. Students were reminded of these objectives before providing informed consent to participate at the start of the focus group. Author B.O. led the focus groups with co-moderation from R.V. Focus groups were scheduled for males and females separately but on the same day, taking place at the University of Waterloo School of Pharmacy. Each focus group lasted approximately 75 min. Both authors kept field notes during the focus groups, and discussions were audio recorded and transcribed verbatim using Rev.com to facilitate analysis. It was determined a priori that only one focus group with each gender would be performed, regardless of whether or not data saturation was achieved.

### 2.5 The research team

The first author (B.Y.O.) was a fourth-year Doctor of Pharmacy (PharmD) student at the University of Waterloo School of Pharmacy at the time of the study, and was therefore conducting the focus group among her peers as part of an Independent Studies research elective course. R.V. is a male sociologist and was a research coordinator holding a Masters degree, who did not have any interaction or relationship with the student participants prior to the focus group. The remaining authors are female faculty members at the University of Waterloo School of Pharmacy and are all pharmacists.

### 2.6 Analysis

Focus group data were coded inductively by two independent reviewers (B.Y.O. and R.V.), employing thematic analysis^16^ using Microsoft Word®, as there were only two transcripts to code (one per focus group). First, each individual reviewed the transcripts independently in full and identified preliminary codes using the highlighting and commenting features within the software. Once these initial codes were identified, transcripts were reviewed in detail between the researchers and highlighted text and associated codes were compared to come up with the coding framework. The transcripts were then re-examined by B.Y.O. using the final coding framework with any uncertainties discussed with R.V. to reach consensus. No additional codes were identified following this discussion. Major themes were defined as those that transcended various focus group questions and prompts and showed agreement across multiple students, with minor themes defined as signals that emerged from the data but were limited to specific situations or were described by a small proportion of participants. Focus group participants did not provide feedback on either the transcript or the identified codes and themes. Results are presented indicating the respondent's gender and transcript line number following any direct quotes.

### 2.7 Ethics

The study received ethics approval from the University of Waterloo Office of Research Ethics (ORE #40447). Participation in the study was voluntary, and participants could withdraw for any reason prior to or during the focus groups.

## 3 Results

Participant demographics are summarized in Table 1. Median wages for first, second, and third co-op placements were all higher among female versus male students. Of 36 unique co-op positions held by students prior to the focus group, students reported engaging in any form of negotiation for only three. Thematic analysis identified three major and two minor themes including: (1) Preservation of the relationship; (2) Power differential and perceived ability to negotiate; (3) Institutional support and training; (4) Negative experience with negotiation and wage gaps; (5) Gendered approach to negotiation.

**Table 1:** Participant demographics and self-reported wages.

	Male (*n* = 7)	Female (*n* = 8)
Cohort		
Class of 2019	2	4
Class of 2020	5	4
Age, years (mean, SD)	24.9 (1.6)	24.6 (1.3)
Wage (mean, SD)		
Co-op #1	$16.19 (4.10), *n* = 7	$16.78 (3.89), *n* = 8
Co-op #2	$16.37 (1.01), *n* = 7	$17.26 (3.48), *n* = 8
Co-op #3	$14.00 (0), *n* = 2	$17.69 (2.13), *n* = 4

### 3.1 Preservation of the relationship

Students appeared to prioritize preserving the relationship with a potential employer over negotiating aspects of the position such as wage, days off, and job duties. Both male and female students spoke about the potential for negotiation of wages to be perceived negatively by employers. When asked whether she felt negotiating may negatively affect her co-op evaluation, one female student stated that she did, because “there's a negative impression that you're pushing for more” (Female, transcript line 511), with another student adding that it may be perceived that “she's just here for the money” (Female, 633). Another student said she felt negotiating would put you “in a negative light” (Female, 535), echoed by a student who said that asking about wage is “harmful to the interview” (Male, 305) because it may contradict how, during the interview, the students focused on the value they place on providing patient care and the learning experience offered.

One male student recalled an experience where he had to speak extensively with management about a salary discrepancy, and he felt that “It definitely damages the rapport, depending on what your relationship was previously… you felt like you were inconveniencing them” (Male, 559).

### 3.2 Power differential and perceived ability to negotiate

The idea of a power dynamic existing between employers and students was a recurring theme in both the male and female discussions. One male student explained that because these employers' references and their evaluations impact students' future jobs/residencies, the employers “have so much power over you...So you tread more cautiously than you may otherwise, just because of the power that they have in their position” (Male, 563). Another participant mentioned that as a student, he felt that they “don't really have a lot of say in anything” (Male, 85) regarding wages in the workplace.

Students' primary concern was receiving a good evaluation, stating that “during co-op, the most important thing is to pass co-op... If you fail your co-op, you're held back a year” (Male, 596) and that “you don't want to negotiate and then get a lower mark which is going to affect your future co-ops” (Female, 505). Securing a position was also important to students, as several male and female students reported that they would feel more comfortable discussing wage once they knew they had secured the co-op position in order to have a “security blanket” (Female, 768). In some instances, students only found out what the salary would be after being matched with and accepting the co-op position.

The short-term academic nature of the position also appeared to influence students' willingness to negotiate. A female student said she does not negotiate wage for co-op positions because she feels “co-op is too short term for me to invest any energy in terms of money” (Female, 513). A male student suggested that many students often “put up with” working conditions that they are not satisfied with because “it's four months” and that they “don't have to go back” if they do not want to (Male, 443).

Both male and female students discussed how logistical factors of the co-op program, such as employer budget and duration of placement, are a limitation to negotiation. For example, one female student said she felt there was no room to negotiate in a co-op position because “with co-op they only budget that certain amount or they only get that amount from the company to hire a student” (Female, 724). A male student said students generally do not negotiate vacation time because they are concerned with fulfilling the 16-week duration required to receive academic credit for the placement.

### 3.3 Institutional support and training

Overall, students felt poorly equipped to engage in negotiation with a potential employer, and lacked confidence in initiating such a conversation. They were also unaware of other students' approaches to interviewing and negotiation. Several male and female students alluded to the fact that it would have been helpful for negotiation to be discussed in classes prior to co-op, with one student saying that if students were more aware of negotiation happening for co-op positions then they would be more willing to discuss it. Of note, negotiation is addressed in a final year professional practice course, with one student commenting that this was helpful, as she “didn't negotiate at all for my co-ops [and] didn't even think about it, didn't know, didn't learn about it” (Female, 696), but expressed greater comfort with it going forward after it was introduced in this final year course.

Students also perceived a role for the School to provide support for students experiencing challenges with potential wage gaps. When male students were posed with a hypothetical scenario where they found out a female colleague was making less money than them, most stated they would feel more comfortable discussing this issue with the school as opposed to the employer directly, due to concerns about possible negative repercussions. One student explained that having the discussion with the school is “more secure than going straight to the employer” (Male, 113).

### 3.4 Minor themes

#### 3.4.1 Negative experience with negotiation and wage gaps

While students expressed not being aware of systemic gender-based wage gaps among their peers, one student expressed a negative experience with addressing other salary-related discrepancies in co-op positions. This student described a time that a group of students, both male and female, working at the same institution, collectively approached management to correct a salary discrepancy. When it was unsuccessful, she said they did not pursue further action because they were “only there for four months, [and] it just wasn't worth it” (Female, 430).

#### 3.4.2 Gendered approach to negotiation

A potential signal of a gender-impacted approach to negotiation was observed. Some female students felt they would be perceived negatively by employers if they were to negotiate, due to negative gender stereotypes about females in the workplace. For example, one student stated that female students “don't want to come off as bossy and they're a little afraid and timid” to negotiate with employers, while male students may be more confident to do so because “they won't seem like a bossy person if they do” (Female, 744). She further explained that female students are “just afraid of how people perceive us… and that's a huge factor with a future employer who's also going to be a future reference who's going to be a boss that we're going to work with” (Female, 744). When negotiation is attempted, styles may also differ by gender, as one female student described that she would use a “tactful” approach to negotiation (Female, 642) while a male student preferred a more direct approach, stating that “If you're going to say it, just say it confidently” (Male, 329).

## 4 Discussion

Pharmacy students rarely engage in negotiation during co-operative hiring processes at the University of Waterloo. Concerns about potentially damaging the relationship with the employer, appearing to be motivated by money rather than the patient care experience, and the short duration of co-op placements appear to primarily influence this lack of interest in negotiation. While evidence of a gender wage gap against female students was not apparent among self-reported wages or students' expressed experiences, evidence of a gender imbalance in willingness to negotiate and negotiating style was suggested, with female students aiming to avoid gendered stereotypes such as being perceived as ‘bossy’ while male students aim to approach negotiation ‘confidently’. Students identified a role for educators in both supporting them and educating them on negotiation, stating that a course activity on negotiation in their final year of studies positively impacted their confidence in future negotiations.

To our knowledge, this is the first study to explore student negotiation and gender-based influence on wages among pharmacy students, precluding comparison against the work of others. However, it appears to be consistent with business and psychology literature suggesting that women may feel inclined to adhere to gender stereotypes of being agreeable, collaborative, and team-oriented versus assertively negotiating for one's personal benefit.^11^ As students in this study reported very rarely negotiating on any of their co-op placements (students reported negotiating for only 3 of 36 positions held to date), we were unable to explore how they approached this type of conversation with potential employers.

A study among male and female surgical residents offers some parallels and additional considerations with our exploratory work.^17^ This study found that female residents reported significantly lower expected and ideal starting salary than male students, were less likely than their male counterparts to strongly agree or agree that they had the tools to negotiate, and were less likely to find the notion of negotiation to be appealing. Nearly two-thirds disagreed that they would be offered a fair salary without needing to negotiate. This suggests that residents anticipate a need for negotiation and believe it is appropriate; however, may lack the skills or desire to. This differs slightly from our participants who did not perceive a need to negotiate for short-term co-op placements, highlighting the need for additional research among pharmacy students nearing graduation and entering their first permanent position.

System-related factors related to the processes around co-op hiring cannot be ignored. Under the current system, employers create and post a job description to a web-based platform. Students review these descriptions and choose which job(s) they wish to apply for. Employers then interview students and rank those that they would be willing to hire in order of preference, and students also rank the jobs they interviewed for by preference. A matching algorithm then decides which students match to which jobs. It is not a requirement for employers to post wage information on the job description, nor to discuss it during the interview; hence, in some situations, students are unaware of their wage until a job has been accepted. When a wage is posted in the job descriptions, students may perceive this as a final offer with the employers only having a finite pot of money set aside to hire a co-op student. As a result, an opportunity to negotiate may not be perceived as possible with these positions. Employers should be encouraged to be aware of this dynamic. To address this, they should consider being explicit in their job descriptions whether there is an opportunity to negotiate by either posting the available wage or indicating that wage will be discussed between the student and employer, and ensuring that wages offered are commensurate with student experience and the current market.

The small sample size of the study, comprising 8 female students and 7 male students, may impact the generalizability of the results across the entire student population or across institutions, and precluded achieving saturation. One may expect that those who volunteered to participate may have come to the focus group with different experiences with negotiation and wage disparities than those who did not volunteer to share their perspectives. Students also indicated that their willingness to negotiate with potential employers for co-op positions was limited by the short-term and academic nature of the placement, so these findings may not translate to experiences related to being hired into permanent positions following graduation. Future research should examine negotiation frequency and styles among students seeking an extracurricular part-time position, and new graduates being hired into their first position as a licensed pharmacist. While our students' wages during co-op placements appeared to show a difference between female and male students (favoring females), this was collected by self-report and may be affected by recall bias. It was outside the scope of this study to confirm this observation using actual wage data collected by the university, or to examine the potential factors that may contribute to such a discrepancy, such as the practice setting (e.g., hospital, community, industrial, etc.) and geographic location of the position. Finally, we acknowledge that all but one member of our research team on this project identify as female, which may have implications related to reflexivity and our interpretation of the data.

## 5 Conclusion

This exploratory study has important implications for pharmacy educators, practitioners, and employers. While wage negotiations related to co-operative education opportunities appear to be rare, students identified a number of factors which influence their ability and desire to negotiate wages during co-op placement including the requirements and logistics of placements, and the perceived power imbalance between students and potential employers. Additionally, when competing against their peers for a position, students want to appear motivated to apply for the position for the patient care experience offered rather than remuneration. Pharmacy students and new graduates also appear to lack knowledge of, and confidence in, using negotiation strategies with potential employers. Pharmacy educators are encouraged to equip students with knowledge and skills related to negotiation early in their training, to ensure negotiation can be effectively applied to positions as pharmacy students and upon entering practice as a pharmacist.

## Funding source

This research was funded through a University of Waterloo HeForShe Gender Equity Research Grant.

## Declaration of Competing Interest

The authors declare that they have no known competing financial interests or personal relationships that could have appeared to influence the work reported in this paper.

## Appendix A Supplementary data

Supplementary material Image 1
