# Supplementary material for: Pharmacy students' perceived willingness and ability to negotiate for paid co-operative education positions
Source: Explor Res Clin Soc Pharm. 2021 May 15;2:100026. doi: 10.1016/j.rcsop.2021.100026 (PMC9030280; doi:10.1016/j.rcsop.2021.100026)
Supplement: Supplementary file 1 — Supplementary material [file mmc1.docx]

Appendix 1. Semi-structured focus group guide

1. Can a few people start by describing a time they did or did not negotiate a job. Why was that? It does not need to be a pharmacy job.
2. For your past pharmacy coop jobs, did you try to negotiate? Why/why not? What did you include in the negotiation (e.g., salary, vacation, job description/tasks)?
3. For those who tried to negotiate, what happened? (Probe/prompt to gather both positive and negative experiences, “Can someone share a positive experience?” “Can someone share a negative experience?”). What did you learn from this experience?
4. How comfortable do you feel negotiating the details of a job? Do you feel confident in your negotiation skills? Why/why not?
5. Have you been taught negotiation approaches? If yes, by whom? (Prompts: in grade school? University? Parents/Family? Partners? Mentors? Specific courses?)
6. Describe the support the School of Pharmacy can provide to help you to learn to negotiate?
7. Have you ever discussed the details of your co-op job (e.g., wage, hours) with other students? If yes, did you perceive any differences related to gender?
